# Mixed neuroendocrine–non-neuroendocrine neoplasm of the ampulla of Vater: a case report

**DOI:** 10.1186/s40792-023-01689-6

**Published:** 2023-06-12

**Authors:** Kenjiro Date, Takaaki Tatsuguchi, Yuzo Shimokawa, Yusuke Niina, Daichi Kitahara, Hirotaka Kuga, Sadafumi Tamiya, Kazuyoshi Nishihara, Toru Nakano

**Affiliations:** 1 Department of Surgery, Kitakyushu Municipal Medical Center, 2-1-1 Bashaku, Kokura Kita-Ku, Kitakyushu, Fukuoka 802-8561 Japan; 2 Department of Gastroenterology, Kitakyushu Municipal Medical Center, 2-1-1 Bashaku, Kokura Kita-Ku, Kitakyushu, Fukuoka Japan; 3 Department of Pathology, Kitakyushu Municipal Medical Center, 2-1-1 Bashaku, Kokura Kita-Ku, Kitakyushu, Fukuoka Japan

**Keywords:** Adenocarcinoma, Ampulla of Vater, Mixed neuroendocrine–non-neuroendocrine neoplasm, Neuroendocrine carcinoma

## Abstract

**Background:**

Mixed neuroendocrine–non-neuroendocrine neoplasms of the ampulla of Vater are rare and heterogenous, making it difficult to achieve a definitive preoperative diagnosis. Herein, we describe a patient in whom a provisional diagnosis of mixed neuroendocrine–non-neuroendocrine neoplasm of the ampulla of Vater was made preoperatively.

**Case presentation:**

Computed tomography revealed an enhancing periampullary tumor in a 69-year-old man with obstructive jaundice. Subsequent duodenoscopy revealed an ulcerated lesion in the swollen ampulla of Vater, from which six biopsies were collected. Pathological examination revealed adenocarcinoma in five of them. The remaining one was a neuroendocrine neoplasm according to immunohistochemical analysis. With a provisional diagnosis of mixed neuroendocrine–non-neuroendocrine neoplasm of the ampulla of Vater, the patient underwent subtotal stomach-preserving pancreaticoduodenectomy with modified Child’s reconstruction and was discharged without complications. Pathological examination revealed both adenocarcinoma and neuroendocrine carcinomas, each accounting for ≥ 30% of the tumor, resulting in a definitive diagnosis of mixed neuroendocrine–non-neuroendocrine neoplasm of the ampulla of Vater. Lymph node metastases with neuroendocrine components were also observed. Adjuvant chemotherapy was not administered because of the patient’s renal dysfunction. Liver and lymph node metastases were detected 2 months after surgery, the neuroendocrine component being considered responsible for that relapse. The patient underwent platinum-based chemotherapy at 50% dosage, which initially resulted in significant tumor shrinkage; however, he died 6 months after surgery.

**Conclusions:**

While these tumors’ heterogeneity make definitive preoperative diagnosis of mixed neuroendocrine–non-neuroendocrine neoplasm of the ampulla of Vater difficult, the possibility of this disease can be considered by careful examination. Further study is needed to establish the optimal diagnostic criteria and treatment strategy.

## Background

According to the latest World Health Organization (WHO) classification, tumors that comprise at least 30% neuroendocrine and 30% non-neuroendocrine cells are defined as mixed neuroendocrine–non-neuroendocrine neoplasms (MiNENs) [1]. MiNENs are rare and most frequently originate in the colon and rectum [2]. MiNENs of the ampulla of Vater (Amp-MiNEN) are particularly rare. Because of their rarity and heterogeneity, optimal treatment strategies and means of obtaining a definitive preoperative diagnosis have not yet been established. Herein, we report a patient in whom a provisional diagnosis of Amp-MiNEN was made preoperatively.

## Case presentation

**Fig. 1:**
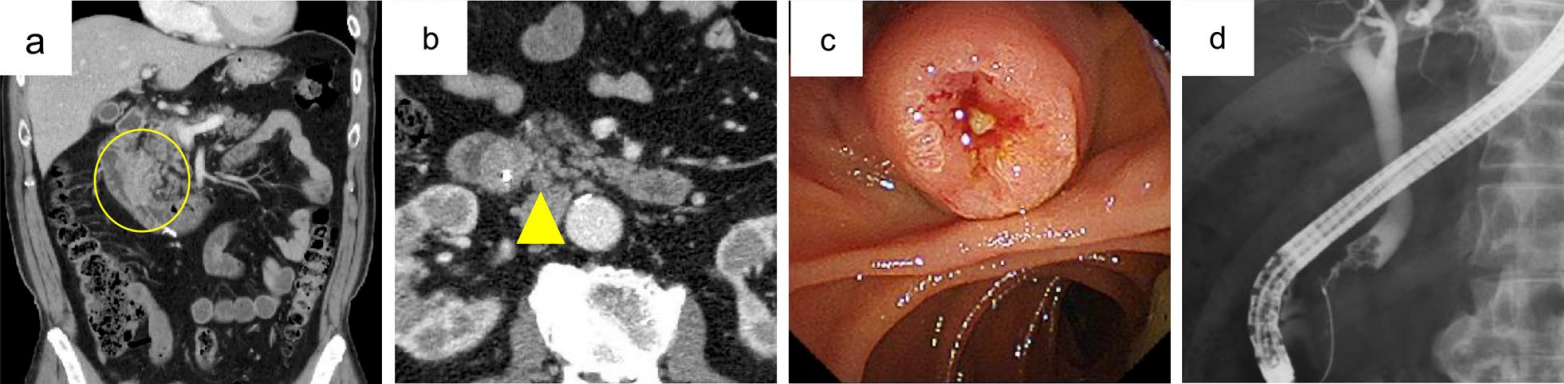
Preoperative examination. **a**, **b** Enhanced computed tomography image showing an enhancing tumor in the ampullary region protruding into the second part of the duodenum (35 mm × 51 mm) (circle) and an enlarged lymph node adjacent to the peripancreatic lesion (arrowhead). **c** Duodenoscopy image showing an ulcerated lesion in the swollen ampulla of Vater. **d** Endoscopic retrograde cholangiography image showing a mass in the distal bile duct arising from the papilla of Vater

**Fig. 2:**
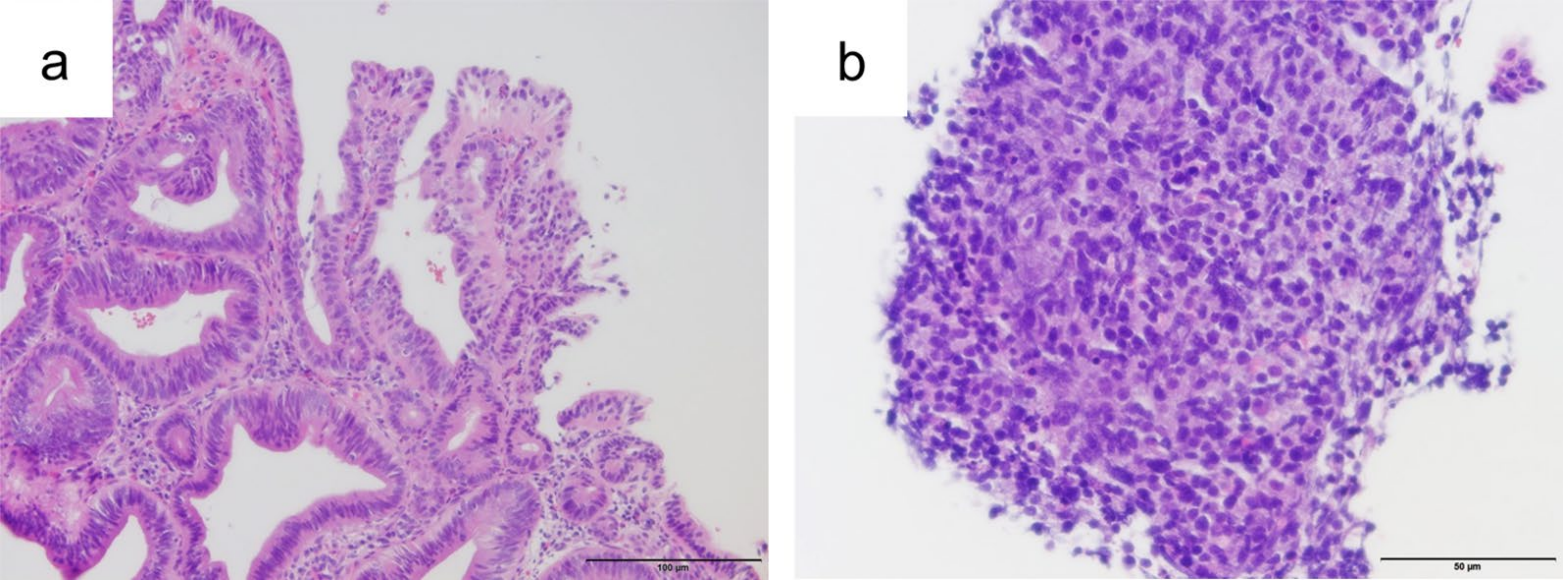
Photomicrographs of biopsy specimens from the ampulla of Vater. **a**, **b** Hematoxylin and eosin stain **a** showing atypical columnar epithelial cells suggestive of adenocarcinoma. Hematoxylin and eosin stain **b** of another specimen showing solid proliferation of epitheloid cells. The tumor cells are positive for chromogranin A and synaptophysin. Scale bars (black lines, bottom right): 100 µm (**a**) and 50 µm (**b**)

A 69-year-old man with a medical history of hypertension and chronic renal failure visited another hospital for jaundice and was suspected of having obstructive jaundice caused by a distal bile duct tumor. Endoscopic retrograde cholangiography and subsequent biliary drainage were performed; however, both bile juice and brushing cytology were negative, thus, precluding a definitive diagnosis. He was, therefore, referred to our hospital for detailed examination and treatment. Laboratory tests revealed anemia, renal dysfunction, and increased concentrations of biliary enzymes and tumor markers: Hb 9.0 g/dL (reference range at our institution 13.7–16.8 g/dL), blood urea nitrogen 36.9 mg/dL (8.0–20.0 mg/dL), creatinine 2.06 mg/dL (0.65–1.07 mg/dL), alkaline phosphatase 158 U/L (38–113 U/L), γ-glutamyl transpeptidase 556 U/L (13–64 U/L), and carbohydrate antigen 19-9 85.9 U/mL (0.0–37.0 U/mL). Enhanced computed tomography showed an enhancing tumor in the ampullary region protruding into the second portion of the duodenum and an enlarged lymph node around the peripancreatic lesion (Fig. 1a, b). Duodenoscopy revealed an ulcerative lesion in the swollen ampulla of Vater (Fig. 1c). Subsequent endoscopic retrograde cholangiography revealed a mass in the distal bile duct arising from the ampulla of Vater (Fig. 1d), and biliary drainage was performed. Bile juice and brushing cytology yielded a diagnosis of adenocarcinoma. Tumor biopsies were also performed to achieve a definitive diagnosis. Pathological examination revealed adenocarcinoma in five of the six specimens obtained (Fig. 2a). The remaining specimen showed proliferation of epitheloid cells that were found by immunohistochemical analysis to be positive for chromogranin A and synaptophysin, consistent with a neuroendocrine neoplasm (Fig. 2b). Based on these findings, an Amp-MiNEN was suspected and the patient referred for curative resection.

**Fig. 3:**
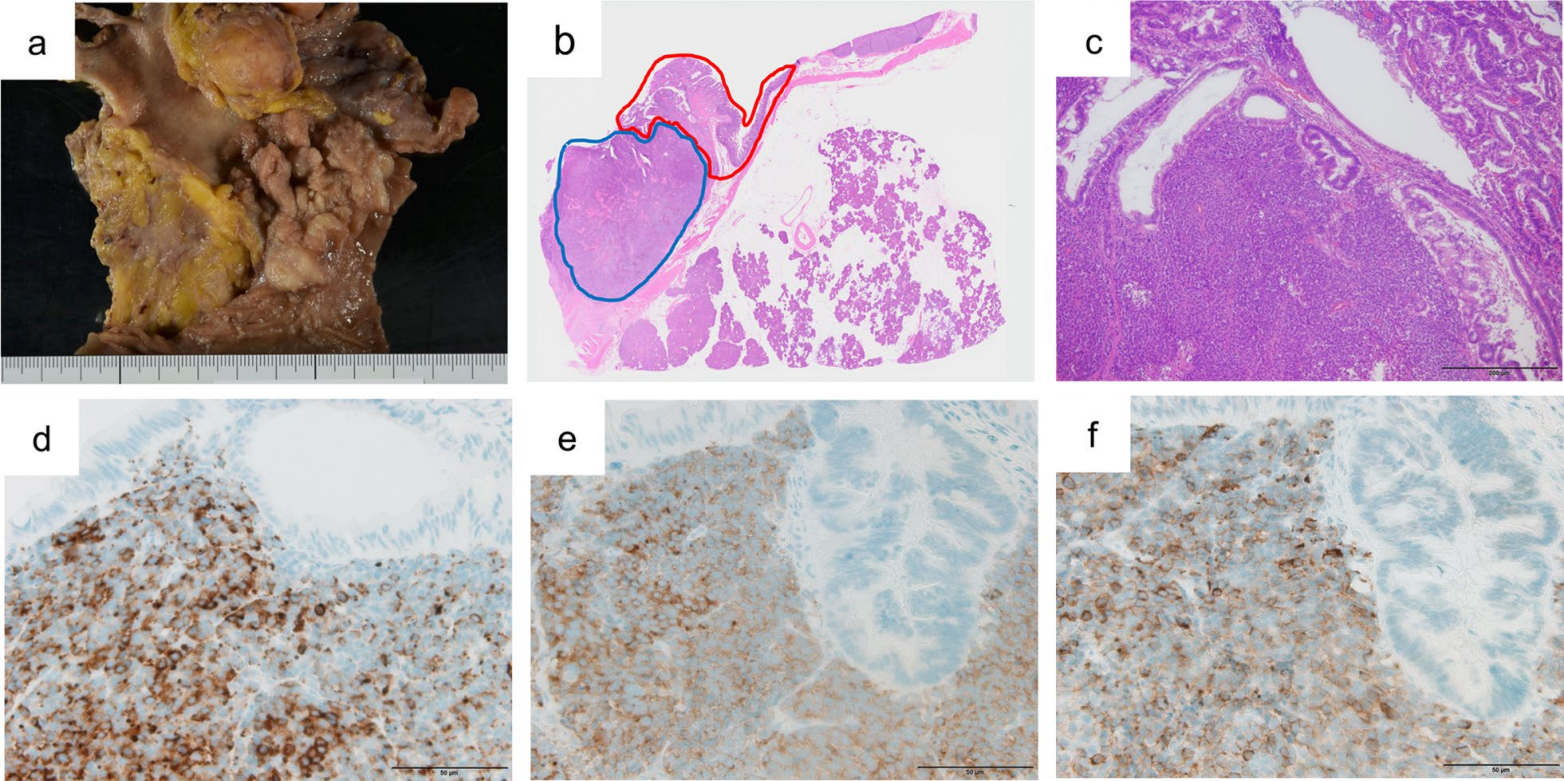
Macroscopic and microscopic findings on examination of the resected specimen. **a** Macroscopic findings. **b** Loupe image. **c**–**f** Microscopic findings. **b**, **c** Hematoxylin and eosin stain. **b** Microscopic findings showing coexisting neuroendocrine (blue area) and adenocarcinoma components (red area). The neuroendocrine component is exposed in the superficial layer in the center of the tumor. **c** The adenocarcinoma components invade neuroendocrine component. **d** Tumor cells are positive for chromogranin A. **e** The tumor cells are positive for synaptophysin. **f** Tumor cells are positive for CD56. Scale bars (black lines, bottom right), 200 µm (**c**), 500 µm (**d**) and 50 µm (**d**–**f**)

At laparotomy, no liver metastases, peritoneal dissemination, or carcinoma cells in peritoneal washings were detected. Subtotal stomach-preserving pancreatoduodenectomy with modified child’s reconstruction and regional lymphadenectomy were performed. Pathological examination revealed that the tumor had both adenocarcinoma and neuroendocrine components (Fig. 3a, b). Each component had its own area and was distributed adjacent to each other. The adenocarcinoma component was basically located in the superficial layer and the neuroendocrine component in the deeper layer. However, in the center of the tumor, the neuroendocrine component was exposed in the superficial layer, which was thought to be resulting from ulceration. There were also areas, where the adenocarcinoma component invaded deeper layer and appeared to be a transition area (Fig. 3c). Immunohistochemical analysis revealed that the neuroendocrine tumor cells were positive for chromogranin A, synaptophysin, and CD56 (Fig. 3d–f). The Mib-1 labeling index was 45.9%, and the neuroendocrine component was poorly differentiated with necrosis. Thus, the neuroendocrine component was diagnosed as neuroendocrine carcinoma (NEC). Tumor cells invading the muscularis propria of the duodenum (T1b[Du]), and lymph node metastases with NEC components were detected (N2, 7/30). The adenocarcinoma and neuroendocrine components each comprised more than 30% of the tumor. The final diagnosis was, therefore, Amp-MiNEN, pT1b(Du)N2M0 fStage IIIB, according to the guidelines of the Japanese Society for Hepato-Biliary-Pancreatic Surgery 7th edition.

The patient was discharged without complications. Adjuvant chemotherapy was not administered because of the patient’s renal dysfunction. Liver and lymph node metastases were detected 2 months after surgery, and he subsequently underwent platinum-based chemotherapy at 50% dosage. This initially achieved remarkable tumor shrinkage; however, he died 6 months after surgery.

## Discussion

In 2010, the WHO classification of gastrointestinal tumors defined tumors containing both adenocarcinoma and NEC as mixed adeno-neuroendocrine carcinomas (MANEC) [3]. However, the term MANEC was an inadequate descriptor for these mixed tumors, because they were not always composed of the two components of adenocarcinoma and NEC. The WHO, therefore, reclassified MANECs as MiNENs, defining these as tumors with neuroendocrine and non-neuroendocrine components, each of which must account for more than 30% of tumor cells [1]. Because the term MiNEN covers a broad spectrum from benign to malignant and these lesions are rare and heterogeneous, treatment strategies and means of obtaining definitive preoperative diagnoses must be decided on a case-by-case basis.

Reaching a definitive preoperative diagnosis of Amp-MiNEN by examining tissue biopsies obtained by duodenoscopy is challenging, because the adenocarcinoma component is characteristically located on the surface. In contrast, the neuroendocrine component is more difficult to access, because it is located in deeper layers: this difficulty commonly resulting in diagnoses of adenocarcinoma [4, 5]. In the present patient, we collected more specimens than usual, because the tumor seemed softer than a typical adenocarcinoma. Based on the pathological findings of the resected specimen and the biopsy results, a biopsy from the ulcer was diagnosed as NEC and biopsies from the ulcer margin were diagnosed as adenocarcinoma. Multiple biopsies yielded a possible diagnosis of Amp-MiNEN. It has been reported that collecting both component with biopsy or cytology is difficult, and in patients with metastatic disease, biopsies are usually performed from metastases, which are likely to have only one component [5–7]. In addition, the requirement for 30% ≥ proportions of each of the major components for a definitive diagnosis of MiNEN is controversial [5–7]. Actually, there have been some reports of mixed tumors that did not meet diagnostic criteria for MiNEN because of this 30% threshold, but can be clinically considered MiNEN [8, 9]. Thus, MiNEN might be under-diagnosed with the current criteria [6], which might lead to difficulty in elucidating the actual epidemiology and prognosis of this disease. Further study is needed to establish the optimal means and criteria for accurately diagnosing this condition.

Given the aggressive nature of MiNENs, multidisciplinary treatment incorporating both radical resection and systemic chemotherapy is indicated [5, 6, 10]. A recent systematic review reported that incomplete resection, Ki-67 index (≥ 50%), disease stage, NEC grade, and non-NEC grade are correlated with poor prognosis; however, no standard treatment has yet been established. Adjuvant therapy may improve the outcome of biliary MiNENs [3]. Tailoring chemotherapy to the more aggressive component has been recommended [4]. The present patient’s NEC component metastasized to the lymph nodes, prompting chemotherapy as post-relapse treatment. The patient had been considered not fit enough for adjuvant chemotherapy because of his renal dysfunction. However, considering the early relapse and subsequent significant response to chemotherapy, even low-dose adjuvant chemotherapy may have been beneficial.

Although the role of neoadjuvant therapy is currently unclear, effort should be put into making a definitive diagnosis if neoadjuvant therapy becomes the standard treatment. Recent remarkable advances in EUS–FNA have achieved greater accuracy in the diagnosis of peripancreatic lesions. If we had used EUS–FNA to assess the enlarged lymph node, we may have realized that the NEC component, rather than the adenocarcinoma, was predominant. Although MiNEN is defined as a single disorder, it is important to recognize that it is a mixed tumor, the components of which behave independently [7]. Because treatment of only one of the two components can lead to progression of the other, careful consideration should be given to administering neoadjuvant therapy. This means that it is important to at least suspect MiNEN preoperatively and to determine which is the predominant component, especially in patients with unresectable disease or for whom neoadjuvant therapy is being considered.

In conclusion, we here report a patient with Amp-MiNEN in whom the correct diagnosis was suspected preoperatively. Although definitive preoperative diagnosis of Amp-MiNEN can be difficult, it can be achieved with careful investigation. Further study is needed to establish the optimal diagnostic criteria and treatment strategy, including perioperative treatment.

## Data Availability

The data sets supporting the conclusions of this article are included within the article.

## Abbreviations

WHO: World Health Organization
MiNEN: Mixed neuroendocrine–non-neuroendocrine neoplasms
Amp-MiNEN: MiNENs of the ampulla of Vater
NEC: Neuroendocrine carcinoma
MANEC: Mixed adeno-neuroendocrine carcinomas
EUS–FNA: Endoscopic ultrasound–fine needle aspiration
